# Differences between the rhizosphere microbiome of *Beta vulgaris* ssp. *maritima*—ancestor of all beet crops—and modern sugar beets

**DOI:** 10.3389/fmicb.2014.00415

**Published:** 2014-08-26

**Authors:** Christin Zachow, Henry Müller, Ralf Tilcher, Gabriele Berg

**Affiliations:** ^1^Austrian Center of Industrial Biotechnology (ACIB GmbH)Graz, Austria; ^2^Institute of Environmental Biotechnology, Graz University of TechnologyGraz, Austria; ^3^KWS SAAT AGEinbeck, Germany

**Keywords:** wild beet, sea beet, sugar beet, rhizosphere, antagonistic bacteria, stress protecting bacteria

## Abstract

The structure and function of the plant microbiome is driven by plant species and prevailing environmental conditions. Effectuated by breeding efforts, modern crops diverge genetically and phenotypically from their wild relatives but little is known about consequences for the associated microbiota. Therefore, we studied bacterial rhizosphere communities associated with the wild beet *B. vulgaris* ssp. *maritima* grown in their natural habitat soil from coastal drift lines (CS) and modern sugar beets (*Beta vulgaris* ssp. *vulgaris*) cultivated in CS and potting soil (PS) under greenhouse conditions. Analysis of 16S rRNA gene fingerprints and pyrosequencing-based amplicon libraries revealed plant genotype- and soil-specific microbiomes. Wild beet plants harbor distinct operational taxonomic units (OTUs) and a more diverse bacterial community than the domesticated sugar beet plants. Although the rhizospheres of both plant genotypes were dominated by *Proteobacteria* and *Planctomycetes*, 37.5% of dominant OTUs were additionally detected in the wild beet rhizosphere. Analysis of the cultivable fraction confirmed these plant genotype-specific differences at functional level. The proportion of isolates displayed *in vitro* activity against phytopathogens was lower for wild beet (≤45.8%) than for sugar beet (≤57.5%). Conversely, active isolates from the wild beet exhibited stronger ability to cope with abiotic stresses. From all samples, active isolates of *Stenotrophomonas rhizophila* were frequently identified. In addition, soil type-specific impacts on the composition of bacterial communities were found: *Acidobacteria*, *Chloroflexi*, and *Planctomycetes* were only detected in plants cultivated in CS; whereas *Bacteroidetes* and *Proteobacteria* dominated in PS. Overall, in comparison to modern sugar beets, wild beets were associated with taxonomically and functionally distinct microbiomes.

## Introduction

The domestication of plants began with the identification of wild plant species exploitable for food, animal feed, or other domestic purposes. Selective crop breeding programs are mainly oriented toward increasing food production and high-yielding varieties, percentage of usable plant parts, and resistance against diseases (Akhalkatsi et al., 2012). The genetic background of sugar beet is narrower compared to other crops because the sugar beet breeding had started in the late eighteenth century when lines accumulating sugar in the storage root were selected from crosses made with chard and fodder beet (Fischer, 1989). In contrast to many other crops, the ancestor of domesticated sugar beets (*Beta vulgaris* ssp. *vulgaris* L.) is known and still distributed throughout Europe, especially in the Mediterranean region along coastal drift lines. The genome of the wild form *B. vulgaris* L. ssp. *maritima* (L.) Arcang. (trivial name: wild or sea beet) differs significantly from domesticated sugar beet lines (Dohm et al., 2014). For example, it shows high variation at the vernalization *B*-locus, and therefore most of the plants are perennials in contrast to all cultivated lines, which have a biennial life cycle enabling seed production (Dohm et al., 2014). Today, modern sugar beet cultivars that accumulate enormous amount of sugar are of importance, not only for commercial sugar production, but also as renewable resource, e.g., for bioethanol production (Dodić et al., 2009). Although several studies analyzing sugar beet-associated bacterial communities already exist (Zachow et al., 2008; Mendes et al., 2011; Shi et al., 2014), little is known about the relation of the microbiome to existing cultivars and their wild relatives.

Plants have recently been recognized as meta-organisms, due to a close symbiotic relationship with their microbiome. Comparable to humans and other eukaryotic hosts, plants also harbor a “second genome” that fulfills important host functions including protection against biotic and abiotic stress (Berendsen et al., 2012; Berg et al., 2013). Interestingly, plant-associated bacteria derived from different origins. Microorganisms can be transmitted by pollen and seeds (Fürnkranz et al., 2012); the latter was also shown for sugar beet (Dent et al., 2004). However, specific microorganisms are enriched from the surrounding environment as well, e.g., attracted by root exudates containing carbohydrates, proteins, and vitamins (Chaparro et al., 2013). In result of these processes, each plant harbors to a certain degree specific microbes (rev. in Berg and Smalla, 2009; Bulgarelli et al., 2012). This specificity was also shown for the plant-associated microbiome at cultivar level, e.g., for rice (Engelhard et al., 2000; Hardoim et al., 2011) as well as for maize (Haichar et al., 2008; Philippot et al., 2013). The evolutionary relationship in wheat–microorganism interactions was revealed already by Germida and Siciliano (2001); ancient wheat cultivars were colonized by phylogenetically diverse rhizobacterial isolates, whereas the rhizosphere of modern cultivars was dominated by fast-growing *Proteobacteria*. Therefore, we developed the hypothesis that the microbiome of the wild beet harbors a high degree of specific microorganisms in comparison to the sugar beet crop. We expected this degree of specificity also for the functions of the associated bacteria. The wild beet plants commonly grow in salinated dune soil of coastal drift lines. Thus, protection against abiotic stress by the microbiome should be more important than against biotic stress because plant diseases are not known for these populations (Biancardi et al., 2012). As reason for the specific microbiome of the wild beet, we assumed not only the genetic background of the wild ancestor; we also hypothesized an impact of the microbiome of the coastal drift line soil.

The objective of this study was: to determine key players in the rhizosphere microbiomes of (i) wild beet plants grown in coastal drift line soil (WB-CS), and (ii) domesticated sugar beets cv. BERETTA (SB) in dependence of the soil type (coastal drift line soil, CS, and potting soil, PS), and (iii) to determine the potential of isolates to cope with biotic and abiotic stresses in successive screenings.

## Materials and methods

### Sampling and experimental design

Wild beet plants (WB) with the synonymous common name sea beet [*B. vulgaris* ssp. *maritima* (L.) Arcang.] were sampled from the drift line at the Mediterranean Sea coast in Slovenia (N 45.590725, E 13.719469). Along a 200 m coastal drift line, four independent samples were taken. On average, plants were 20 cm high. Leaves were unfolded and separately spread on the beet heads without flower buds or seed development. Additionally, we collected coastal drift line soil (CS, pH 9.5, electric conductance S = 1728 μS cm^−1^) from the layer of 0–10 cm depth. Samples were placed into sterile plastic bags and transported to the laboratory at 4°C. Commercial sugar beet (SB) seeds cv. BERETTA KWS were provided by KWS SAAT AG (Einbeck, Germany). Seeds (10 per pot) were planted in a potting bulk soil (PS, soil:sand:vermiculite mixture 3:1:1, potting soil basis: “Gramoflor Profi-Substrat-Topfpikier M+Ton+Fe,” GBC, Kalsdorf, Austria, pH (CaCl_2_) 5.8, electric conductance S = 1330 μS cm^−1^, containing white and black peat with 90 kg m^−3^ moist clay, 1.0 kg m^−3^ PG-Mix (Greenworld, Wels, Austria), 50 g m^−3^ Radigen® (Terraflor, Iserlohn, Germany); sand: “Maxs Spielsand®,” Scherf GmbH & Co KG, Hartberg, Austria; vermiculite: “Verm 3–6 mm,” Ratioform, Vienna, Austria) as well as in coastal bulk soil from the drift line (CS). For SB-PS, five replicates, and for CS—due to the limited amount of available soil and plant material—three (SB-CS) replicates were incubated under greenhouse conditions (12 h light/dark at 18°C, 60% humidity) for 2 weeks. To isolate microorganisms, 20 g of roots with adhering soil were mixed with 50 ml sterile 0.85% NaCl for 3 min in a laboratory blender (BagMixer, Interscience, Mourjou, France). Suspensions were used for cultivation-independent and -dependent analyses. Samples were labeled as follows: CS—coastal drift line soil, PS—potting soil, SB-CS—sugar beet plants cultivated in coastal drift line soil, SB-PS—sugar beet plants cultivated in potting soil, WB-CS—and wild beet plants grown in coastal drift line soil.

### Molecular fingerprints of 16S rRNA genes

For cultivation-independent analyses, 8 ml of the above mentioned suspension were centrifuged at high speed (16,000 × g, 4°C) for 20 min and the resulting microbial pellets were stored at −70°C until further processing. Total DNA of the rhizosphere communities were extracted by mechanical disruption and homogenization of the pellet using FastDNA Spin Kit for Soil and a FastPrep Instrument (MP Biomedicals, Illkirch, France) for 30 s at 5.0 m s^−1^. DNA was additionally purified by the GeneClean Turbo Kit (MP Biomedicals, Illkirch, France) containing guanidine thiocyanate to remove humic acids. Extracted DNA was treated with RNase (0.02 ng μl^−1^) for 5 min at 65°C to obtain the template for PCR amplification of 16S rRNA genes from total community DNA.

Single strand conformation polymorphism (SSCP) analysis was carried out according to Schwieger and Tebbe (1998) using a 8.0% polyacrylamide gel running 26 h for bacterial community analysis at 400 V. SSCP-PCR was performed according to Köberl et al. (2011). Gels were transmissively scanned (Epson perfection 4990 Photo, Nagano, Japan) to obtain digitized gel images. Normalization and cluster analysis of band patterns, evaluated on band intensity, were carried out with the GelCompar II program (Applied Maths, Sint-Martens-Latem, Belgium). The cluster analysis was performed using the following settings: dendrogram type: unweighted pair group method with arithmetic mean (UPGMA); curve based similarity coefficient: Pearson correlation; optimization 4%, and position tolerances 1%. Background correction was applied for each track. The Pearson correlation index for each pair of lanes within a gel was calculated as a measure of similarity between the community fingerprints.

### Amplicon pyrosequencing and bioinformatic analysis

The hypervariable V4-V5 region of the 16S rRNA gene (*Escherichia coli* positions 515–927) was amplified in a nested PCR approach for 454 pyrosequencing to analyze the taxonomic composition of the bacterial rhizosphere community. The first PCR was conducted with the primer pair 27f/1492r (Lane, 1991), while the second PCR targeted the V4-V5 region with the primer set that contained the 454 pyrosequencing adaptors and sample- specific tags (Table S1). The reaction mixture for the first PCR (10 μl) contained 1 × Taq&Go (MP Biomedicals, Eschwege, Germany), 0.1 μM of each primer and 1 μl of template DNA (95°C for 5 min, 30 cycles of 95°C for 30 s, 57°C for 30 s, 72°C for 90 s, and elongation at 72°C for 5 min). The second PCR (60 μl) was performed by using 1 × Taq&Go, 1.5 mM MgCl_2_, 0.1 μM of each primer and 3 μl of first PCR template (95°C for 5 min, 32 cycles of 95°C for 20 s, 54°C for 15 s, 72°C for 30 s, and elongation at 72°C for 10 min). PCR products of four replicates per samples of the same habitat were pooled in equal molarities and purified with the Wizard™ SV Gel and PCR Clean-Up System (Promega, Madison, USA). 16S rRNA gene amplicons were pyrosequenced with Roche 454 FLX GS conducted by MWG Biotech (Ebersberg, Germany). De-multiplexed raw sequences were processed using the open source software package QIIME (release 1.8.0; Caporaso et al., 2010). Prior to denoising (Reeder and Knight, 2010) sequences were quality (minimum average quality score in reads: 25) and length filtered (430–450 bp). Chimeric sequences were detected via ChimeraSlayer and subsequently removed. Chimera check was followed by excluding plant-originated plastid sequences using BLASTn algorithm (Altschul et al., 1990). Remaining sequences were clustered at 97% similarity using the UCLUST algorithm (Edgar, 2010) and taxonomic assignment of representative sequences were performed using the RDP naïve Bayesian rRNA classifier (Wang et al., 2007) based on the reference database Greengenes release 13_5 (De Santis et al., 2006). Read number per sample was normalized to 5578. Rarefaction analysis (species level at 97%), richness estimates, and diversity indices were calculated; Shannon (1997) and Chao1 (Chao and Bunge, 2002) indices were calculated based on the complete linkage clustering data. For OTU-based analysis, only OTUs accounting for at least 1% of total reads were considered. Classifications of the reads (one representative per OTU) were performed by manual alignment of representative sequences with 16S rRNA reference gene sequences from NCBI database using BLASTn algorithm. Raw pyrosequencing data were deposited at the National Center for Biotechnology Information under the BioProject number PRJNA233435 with the SRA accession numbers SRX652486 (WB-CS), SRX652836 (CS), SRX652838 (SB-CS), SRX652839 (SB-PS), SRX652840 (PS).

### Isolation and characterization of bacteria

The homogenized suspensions of wild beet rhizosphere replicates were used for dilution and plating on R2A (Roth, Karlsruhe, Germany), and Kings B amended with ampicillin (50 μg ml^−1^), novobiocin (45 μg ml^−1^), and cycloheximide (50 μg ml^−1^). Each dilution was plated in duplicates. Plates were incubated for 5 days at room temperature (RT) and colony forming units (CFU) were counted to calculate the means of colonies (log_10_ CFU) based on fresh weight (fw). To obtain the microbial communities of commercial sugar beet plants (SB-CS, SB-PS), 2 weeks after germination the culturable fraction of the rhizospheres were harvested, suspended, diluted, and plated as described above. If possible, 24 bacterial isolates were selected randomly for each replicate and subsequently cultured on nutrient agar (NA). Due to the limited amount of CS, fewer replicates were available and therefore, a lower number of bacteria were isolated. The isolates were purified and then stored at −70°C in nutrient broth II (NB II) (Sifin, Berlin, Germany) stocks containing 15% (v/v) glycerol. Isolates were encoded using a combination of letters and numbers indicating: (1) plant/soil type (WB—wild beet plants grown in coastal drift line soil WB-CS, VN—sugar beet plants cultivated in coastal drift line soil, VS—sugar beet plants cultivated in potting soil), (2) replicate (1–4), (3) consecutive number of the isolate per replicate, and (4) origin of the medium (no further indication—bacteria from R2A, Ps—*Pseudomonadaceae* from Kings B). Bacteria with the highest antagonistic activity and stress tolerance were identified by sequencing partially the 16S rRNA gene using the primer pair 27f/1492r (Lane, 1991). Purified fragments were sequenced using LGC Genomics GmbH sequencing service (Berlin, Germany) and identified as described. Sequences obtained were submitted to EMBL Nucleotide Sequence Database under accession numbers KJ024636 to KJ024701. Co-occurrence of 16S rRNA genes from isolates and OTUs obtained by pyrosequencing was determined by aligning corresponding regions using BLASTn algorithm.

### Screening for bacteria antagonistic toward plant pathogens

The antagonistic potential of randomly selected isolates was assessed by *in vitro* inhibition of sugar beet-pathogenic fungi *Alternaria alternata* Nees, *Botrytis cinerea* Persoon, *Rhizoctonia solani* Kühn AG2-2IIIB, *Sclerotinia sclerotiorum* Fuckel, and *Verticillium dahliae* Klebahn V25 according to Berg et al. (2002). Zones of inhibition were measured 3–7 days after incubation at 25°C.

### Screening for stress tolerating bacteria

Overnight cultures grown in 10 ml NBII were used as inoculum (5 μl) for all bacterial assays. For desiccation assays, 20 μl of culture fluids were dried under sterile conditions. After 3, 6, 9, 16, 21, and 56 days, bacteria were re-suspended with the same volume NBII and dropped on NA. Re-cultivated cells were registered in a positive/negative response. For osmolarity stress, bacteria were cultivated in a 96-well plate filled with 145 μl per well modified Luria Bertani (per liter: peptone 3 g, meat extract 5 g) with various sodium chloride concentrations (1% steps, 0–20%) and incubated at 30°C for 48 h under agitation. In reactive oxygen species stress assays, bacteria were cultivated in NBII amended with different tellurite (1, 3, 5, 7, and 9 mg ml^−1^) and H_2_O_2_ (0.1, 0.3, 0.5, 0.7, and 0.9 mM) concentrations. The assays were performed in 96-well-plates filled with 195 μl medium per well and 5 μl 1:100 diluted overnight incubated culture. Optical density (OD) was measured for all 96-well plates at 600 nm. Bacterial growth was positively evaluated when OD_600_ was higher than 0.2 (that is 5-fold higher than the untreated control) after 24 h.

### Statistics

Significant differences of 16S rRNA gene based fingerprints between all samples were calculated with permutation analysis of pairwise similarities using permtest package for R statistics (The R Foundation for Statistical Computing Version 2.1.1) (Kropf et al., 2004). Other significant differences were calculated using ANOVA with *post-hoc* test Scheffé in SPSS-PASW Statistics v.18.

## Results

### Molecular fingerprints of 16S rRNA genes of the bacterial communities

**Figure 1:**
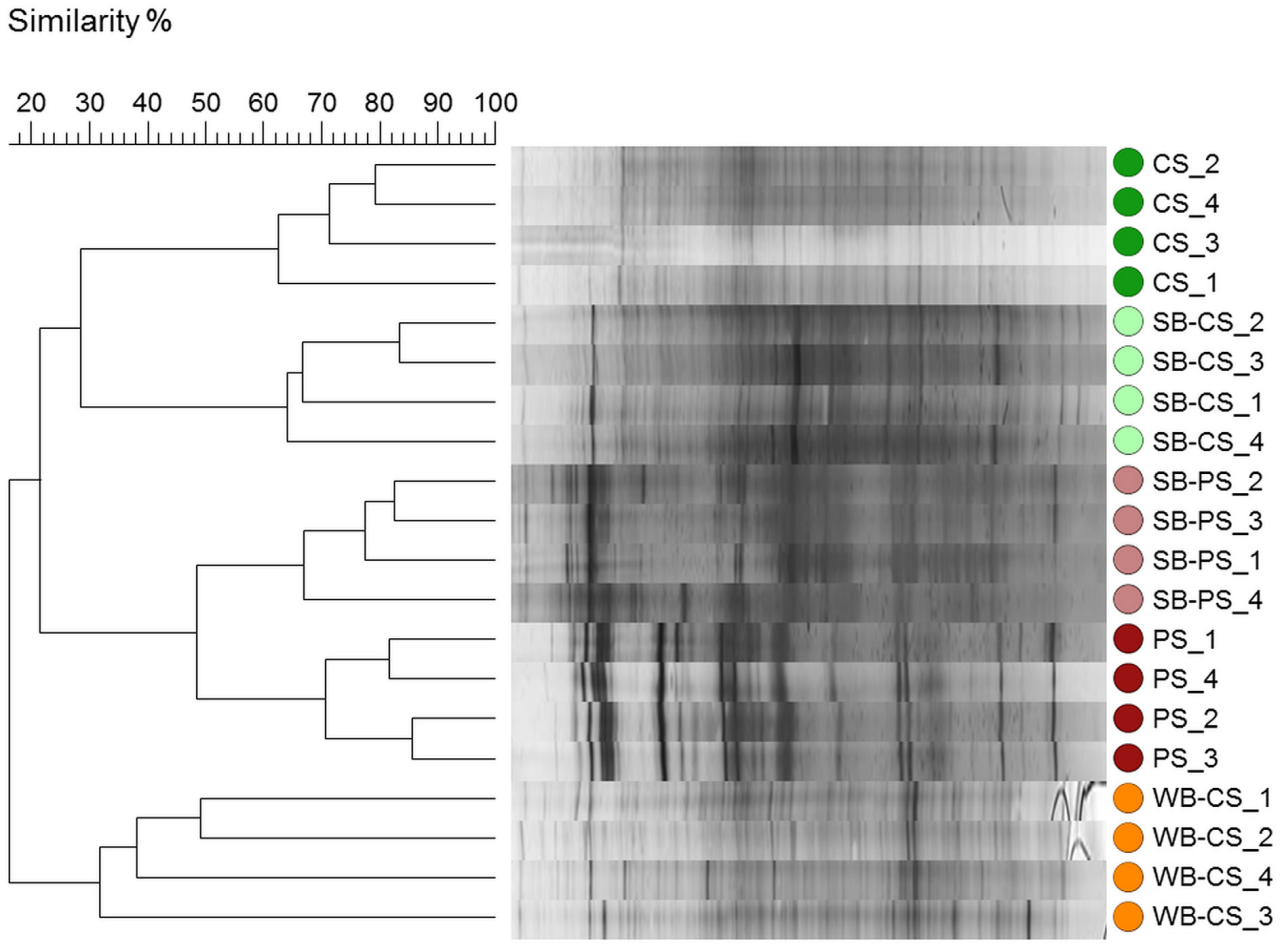
**Dendrogram of 16S rRNA gene-based SSCP profiles of bacterial communities in association with wild beet (WB-CS) and sugar beet rhizosphere grown in coastal drift line (SB-CS) and potting soil (SB-PS) and their respective bulk soils (CS, PS) with four repetitions per sample**. Following settings were used: dendrogram type: unweighted pair group method with arithmetic mean (UPGMA); similarity coefficient: curve based, Pearson correlation; optimization: 4%, position tolerance: 1%.

Molecular fingerprints were performed by SSCP analysis of 16S rRNA genes amplified from DNA obtained from all samples to gain first insight into the bacterial communities. Bacterial fingerprints of potting soil samples (SB-PS and PS) comprised fewer but more dominant bands compared to the SSCP profiles from WB-CS, SB-CS, and CS, where a higher number of SSCP bands were detected (Figure S1). According to cluster analyses, bacterial communities from SB-CS and CS significantly differed by 78% from SB-PS and PS (*P* ≤ 0.001), which clearly indicated the high impact of soil type and the composition of bacterial fingerprints. In addition, the rhizosphere effect was highly pronounced: SB-CS and SB-PS were significantly different from their respective bulk soils CS and PS by 72% (*P* ≤ 0.029) and 52% (*P* ≤ 0.028), respectively, (Figure 1). Differences between both investigated plant genotypes were also identified: the WB-CS rhizosphere was separated from all others and showed only 16% similarity with the SB-CS rhizosphere. Noticeably, due to different life cycles not only the genotype but also the plant developmental stage were different and could account for observed effects.

### Pyrosequencing-based 16S rRNA profiling of the bacterial communities

A pyrosequencing-based analysis of partial 16S rRNA gene sequences was employed to survey the diversity and composition of the bacterial rhizosphere communities in WB-CS, SB-CS and SB-PS, and the respective bulk soils CS and PS. In all samples, we recovered between 5578 and 9567 quality sequences with read lengths ranging from 430 to 450 bp. Prior to further analysis, read numbers were normalized to 5578 for each sample. To calculate rarefaction curves and to perform taxonomic assignments, reads were clustered in operational taxonomic units (OTUs) at sequence divergences of 3% (species level). The rarefaction curves of the bacterial communities of WB-CS, SB-CS, SB-PS, and their respective bulk soils CS and PS are shown in Figure S2. At a dissimilarity level of 3%, the curves of WB-CS and PS samples generally showed low slopes, but did not reach saturation. Accordingly, the number of observed OTUs covers only 31.4 and 34.5% of the estimated taxonomic richness by the Chao1 richness estimator (Table 1). That indicates evenly contributing species and a low number of very common or very rare species. The computed Shannon indices of diversity (H') were much higher for the wild beet plants grown in coastal drift line soil (WB-CS) and the sugar beets grown therein SB-CS, and CS (8.7, 8.1, 8.2) than for SB-PS and PS (6.0, 4.3).

**Table 1:** **Species richness (normalized at 5578 sequences per sample) estimates obtained at 3% genetic dissimilarity from 454 pyrosequencing-derived sequences of DNA extracted from SB-CS—Sugar beet plants cultivated in coastal drift line soil, SB-PS—Sugar beet plants cultivated in potting soil, WB-CS—Wild beet plants grown in coastal drift line soil, CS—Coastal drift line soil, PS—Potting bulk soil**.

**Sample ID**	**Sample**	**Shannon index^a^ (H′)**	**Rarefaction^b^ (no. of OTUs)**	**Chao1^c^ (no. of OTUs)**	**Coverage (%)**
MID1	WB-CS	8.7	1369.9	4356.9	40.6
MID2	CS	8.2	1156.1	3153.2	36.7
MID3	SB-CS	8.1	1190.7	2930.2	36.7
MID4	SB-PS	6.0	361.5	986.2	34.5
MID5	PS	4.3	121.3	351.1	31.4

a higher number indicates more diversity;

b results from the rarefaction analyses;

c non-parametric richness estimator based on the distribution of singletons and doubletons.

**Figure 2:**
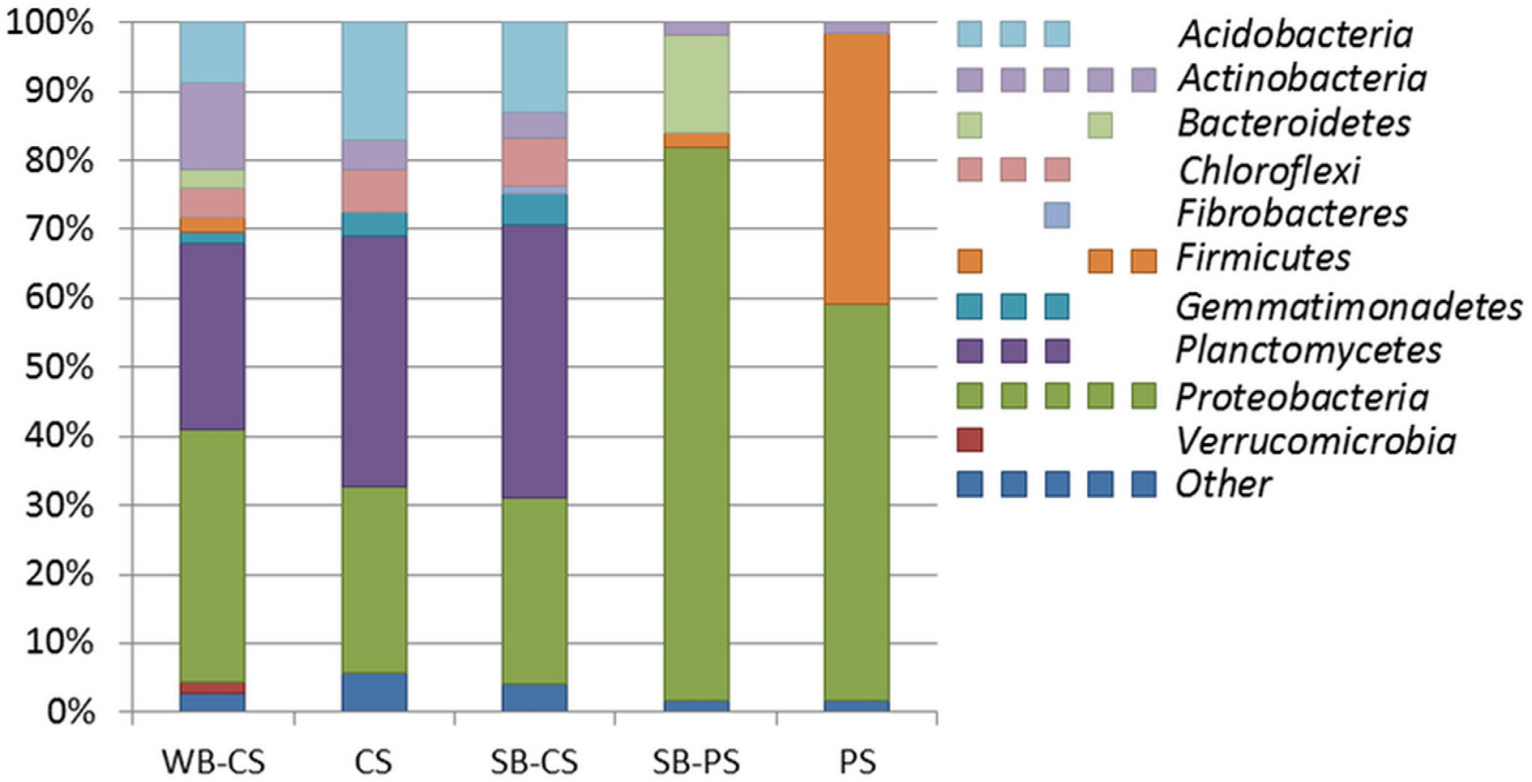
**The structure of bacterial communities at phylum level of wild beet rhizosphere (WB-CS), coastal drift line bulk soil (CS), sugar beet plants cultivated in coastal drift line soil (SB-CS), sugar beet plants cultivated in potting soil (SB-PS), and potting bulk soil (PS)**. Relative composition of major phyla (>1% of total reads) was determined by 454 amplicon pyrosequencing of 16S rRNA extracted from all samples. Multi-colored charts at the legend are shown for each phylum and sample correspondingly. Phyla to which <1% of reads were assigned were summarized as “other.”

Altogether, 98.8% of the OTUs (total number: 5163) were affiliated to 10 different phyla representing at least 1% of reads (Figure 2): *Actinobacteria* and *Proteobacteria* were found in all samples. Only in WB-CS, SB-CS, and CS *Acidobacteria* (8.7, 13.0, 17.1%), *Chloroflexi* (4.3, 7.0, 6.0%), *Gemmatimonadetes* (1.6, 4.7, 3.7%), and *Planctomycetes* (26.9, 39.3, 36.3%) were found. *Bacteroidetes* were found exclusively in rhizospheres of WB-CS (2.7%) and SB-PS (14.1%). Low percentages of *Fibrobacteres* were found exclusively in the rhizosphere of SB-CS (1.0%) and *Verrucomicrobia* in the rhizosphere of WB-CS (1.7%).

### Soil type- and plant genotype-specific OTUs

A more detailed analysis of the dominant soil and plant genotype-specific OTUs was performed on the base of a manual taxonomic assignment by BLASTn alignment using NCBIs 16S rRNA gene reference database. In total, 48 OTUs were obtained when a cut-off level of 1% was applied (Table 2). The dominant genus within the phylum *Proteobacteria* was *Pseudomonas* comprising four species (OTU1726, 2094, 2281, 4102) with the relative abundance of 18.8% detected in the SB-PS rhizosphere, 1.8% WB-CS rhizosphere, 0.2% in the SB-CS rhizosphere, and 16.9 and 0.1% in PS and CS, respectively. The second major genus assigned to *Stenotrophomonas* comprised only one OTU (3731), and was detected in the SB-PS (1.8%) and WB-CS (0.4%) rhizosphere, and in PS (21.1%). Most of the *Firmicutes* sequences belonged to the genus *Paenibacillus* comprising three species (OTU1033, 2251, 3065) in SB-PS (0.2%) and PS (8.3%), followed by *Bacillus* (OTU379) in the SB-PS (0.2%) and WB-CS rhizosphere (0.1%), and in PS (6.7%). *Blastopirellula*, the main genus within the phylum *Planctomycetes*, occurred in approximately equal abundances in the SB-CS (4.9%) and WB-CS (4.0%) rhizosphere, and in CS (5.2%). The majority of *Bacteroidetes* reads belonged to *Flavobacterium* (OTU254, 3902) and *Pedobacter* (OTU172, 3215) detected in the SB-PS and WB-CS rhizosphere. Within the *Actinobacteria*, *Thermoleophilum* occurred as the only genus (OTU745) in the SB-CS (3.1%) and WB-CS rhizosphere (0.6%), and CS (2.5%). Within the *Acidobacteria* the OTU945 assigned to *Candidatus Solibacter* occurred with 0.7% in SB-CS, 0.2% WB-CS, and 1.3% in CS.

**Table 2:**
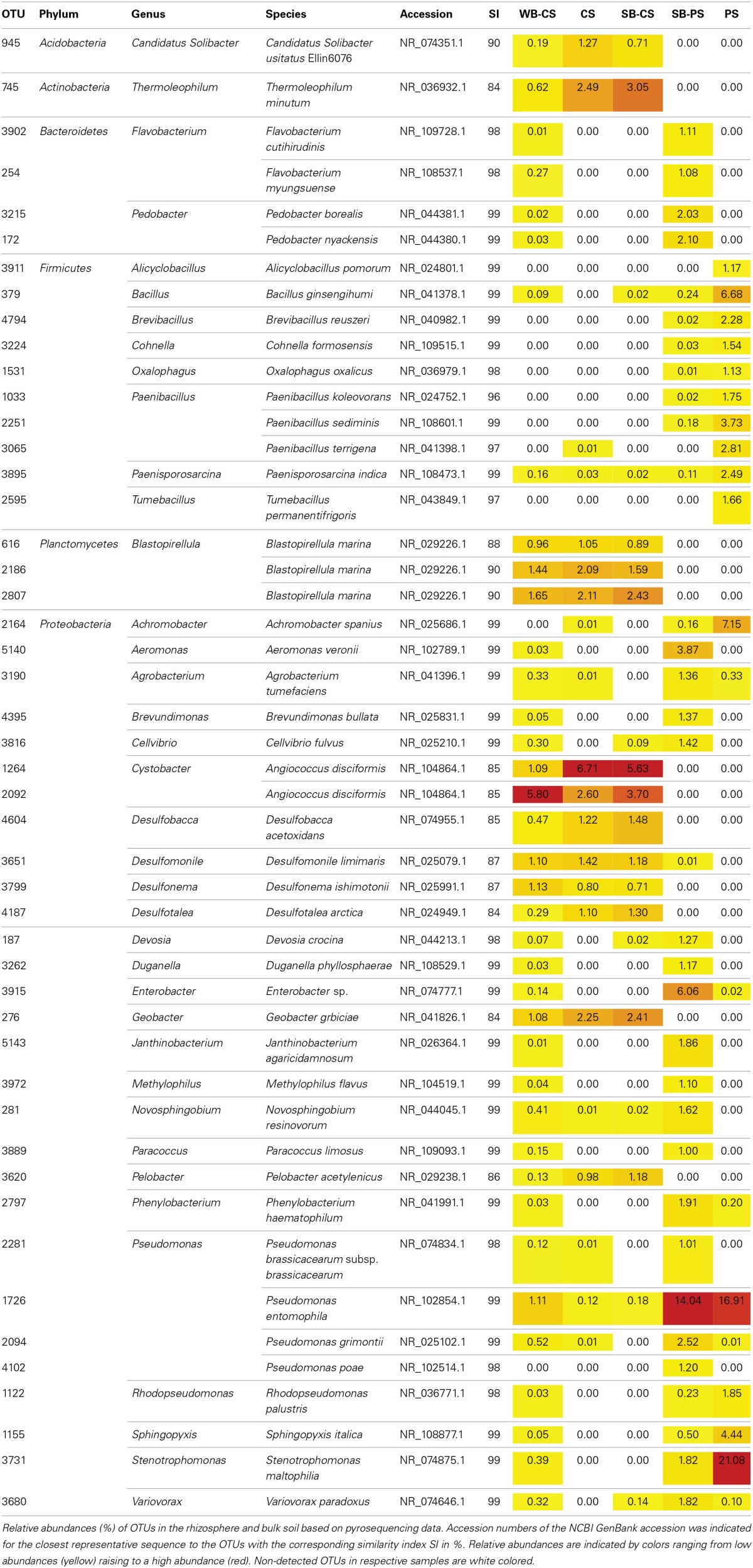
**Taxonomic classification and relative abundance of dominant OTUs (cut-off level: 1%) from rhizosphere and soil samples**.

Relative abundances (%) of OTUs in the rhizosphere and bulk soil based on pyrosequencing data. Accession numbers of the NCBI GenBank accession was indicated for the closest representative sequence to the OTUs with the corresponding similarity index SI in %. Relative abundances are indicated by colors ranging from low abundances (yellow) raising to a high abundance (red). Non-detected OTUs in respective samples are white colored.

Comparing wild beet and sugar beet cultivated in CS, 20 out of 38 dominant OTUs (41.7%) were detected in both rhizospheres, whereas 18 OTUs (37.5%) accounting for 2.3% of total reads occurred exclusively in WB-CS, i.e., OTU172, 254, 1122, 1155, 2094, 2281, 2797, 3190, 3215, 3262, 3731, 3889, 3902, 3915, 3972, 4395, 5140, 5143 (Table 2). The WB-CS-specific OTU with the highest percentage of reads (0.5%) was OTU2094 assigned to *Pseudomonas grimontii*. Remarkably, none of the OTUs was found to be specific for SB-CS. In the soil type comparison of sugar beet plants cultivated in CS and PS, 12 specific OTUs (25.1% of total reads) were found in SB-CS and 25 in SB-PS (33.7%). In SB-CS, the most dominant and specific OTU (5.6%) was assigned to *Angiococcus disciformis* (OTU1264) and in SB-PS to *Enterobacter* sp. with 6.1% (OTU3915). Altogether, a high impact of soil type and also of plant genotype on the rhizosphere communities was observed.

### Comparative analyses of rhizosphere and bulk soil responders

Taxonomic comparisons were done at genus level for representative sequences of all OTUs obtained by clustering at 97% similarity. Classification was performed using the RDP naïve Bayesian rRNA classifier and Greengenes 16S rRNA database. Overall, 37.4% of the sequences could be assigned to 70 genera with a confidence score of ≥80% (Table S2). By comparing CS and WB-CS, 44 taxa assigned to eight phyla were detected (including *Other*). In general, 31 taxa responded positively in the rhizosphere with increased ratio higher than 2, where the highest ratio was found for *Novosphingobium* sp. (74.4) followed by *Pseudomonas* sp. (50.8). Comparing CS and SB-CS, only six taxa (ratio > 2) were positive rhizosphere responders with the highest ratio found for *Cellvibrio* sp. (6.1) followed by *Aquimonas* sp. (4.3); whereas 32 taxa were not detected at all in CS. By comparing PS and SB-PS seven taxa (ratio > 2) were detected with the highest ratio of 116.0 found in the rhizosphere and assigned to *Sphingomonas* sp. followed by *Pseudomonas* sp. (90.6). Fifty-two taxa were not detected in PS. For wild beet plants in CS and sugar beet plants cultivated in CS and PS, the rhizosphere effect could be confirmed. In general, a stronger rhizosphere effect was observed for sugar beet plants in potting soil compared to plants cultivated in coastal drift line soil.

### Bacterial antagonists and their stress response

In total, 576 bacteria were isolated; 192 isolates were selected from WB-CS, 144 from SB-CS, and 312 from SB-PS rhizospheres. The lower number of isolates from WB-CS and SB-CS resulted from the limited amount of sample material. All bacteria were tested in dual culture assays for their *in vitro* antagonistic activity against various sugar beet phytopathogens. In total, 357 bacteria were positive against at least one of the phytopathogens. The highest proportion of antagonistic bacteria originated from Kings B agar compared to R2A (Table 3).

**Table 3:** **Antagonistic potential toward various plant pathogens**.

**Origin**	**Cultivation medium**	**No. of isolates^a^**	***A. a.* (%)**	***B. c.* (%)**	***R. s.* (%)**	***S. s.* (%)**	***V. d.* (%)**
WB-CS	R2A	96	7.3 ± 10.2 a	4.2 ± 7.1 a	0.0 ± 0.0 a	2.1 ± 3.5 a	2.1 ± 3.5 a
SB-CS	R2A	72	12.5 ± 3.3 a	10.4 ± 8.9 a	2.1 ± 3.5 a	2.1 ± 3.5 a	12.5 ± 16.8 b
SB-PS	R2A	120	1.7 ± 2.1 a	15.0 ± 9.7 a	0.8 ± 1.5 a	1.7 ± 2.1 a	n.d.
WB-CS	Kings B	96	17.7 ± 15.6 a	45.8 ± 20.2 ab	10.4 ± 8.9 a	1.0 ± 1.8 a	4.2 ± 2.9 a
SB-CS	Kings B	72	20.8 ± 8.2 a	27.1 ± 3.5 a	4.2 ± 7.1 a	16.7 ± 11.5 b	20.8 ± 7.1 b
SB-PS	Kings B	120	10.1 ± 5.5 a	57.5 ± 11.6 b	4.6 ± 2.5 a	n.d.	n.d.

Legend: SB-CS—Sugar beet plants cultivated in coastal drift line soil, SB-PS—Sugar beet plants cultivated in potting soil, WB-CS—Wild beet plants grown in coastal drift line soil. Inhibition of typical and new emerging sugar beet phytopathogenic fungi A. a.—Alternaria alternata Nees, B. c.—Botrytis cinerea Pers., R. s.—Rhizoctonia solani Kühn AG2-2IIIB, S. s.—Sclerotinia sclerotiorum Fuckel, and V. d.—Verticillium dahliae V25. n.d.—not determined.

a The lower number of isolates from WB-CS and SB-CS resulted from the limited amount of sample material.

The highest percentage of antagonists were found for bacteria isolated from SB-PS (R2A 15.0 ± 9.7, Kings B 57.5 ± 11.6) against *Botrytis cinerea* independently from the selection medium. Within isolates from R2A, a higher percentage of active bacteria were found from SB-CS compared to WB-CS except for *Sclerotinia sclerotiorum*. Similarly, for bacteria isolated from Kings B, a higher percentage of active bacteria were isolated from SB-CS compared to WB-CS against *A. alternata*, *S. sclerotiorum*, and *V. dahliae*, except for *B. cinerea* and *R. solani*. When cultivated in different soil types, isolates from SB-PS (Kings B) showed a higher antagonistic percentage against *B. cinerea* and *R. solani* and less activity against *A. alternata* compared to SB-CS.

**Figure 3:**
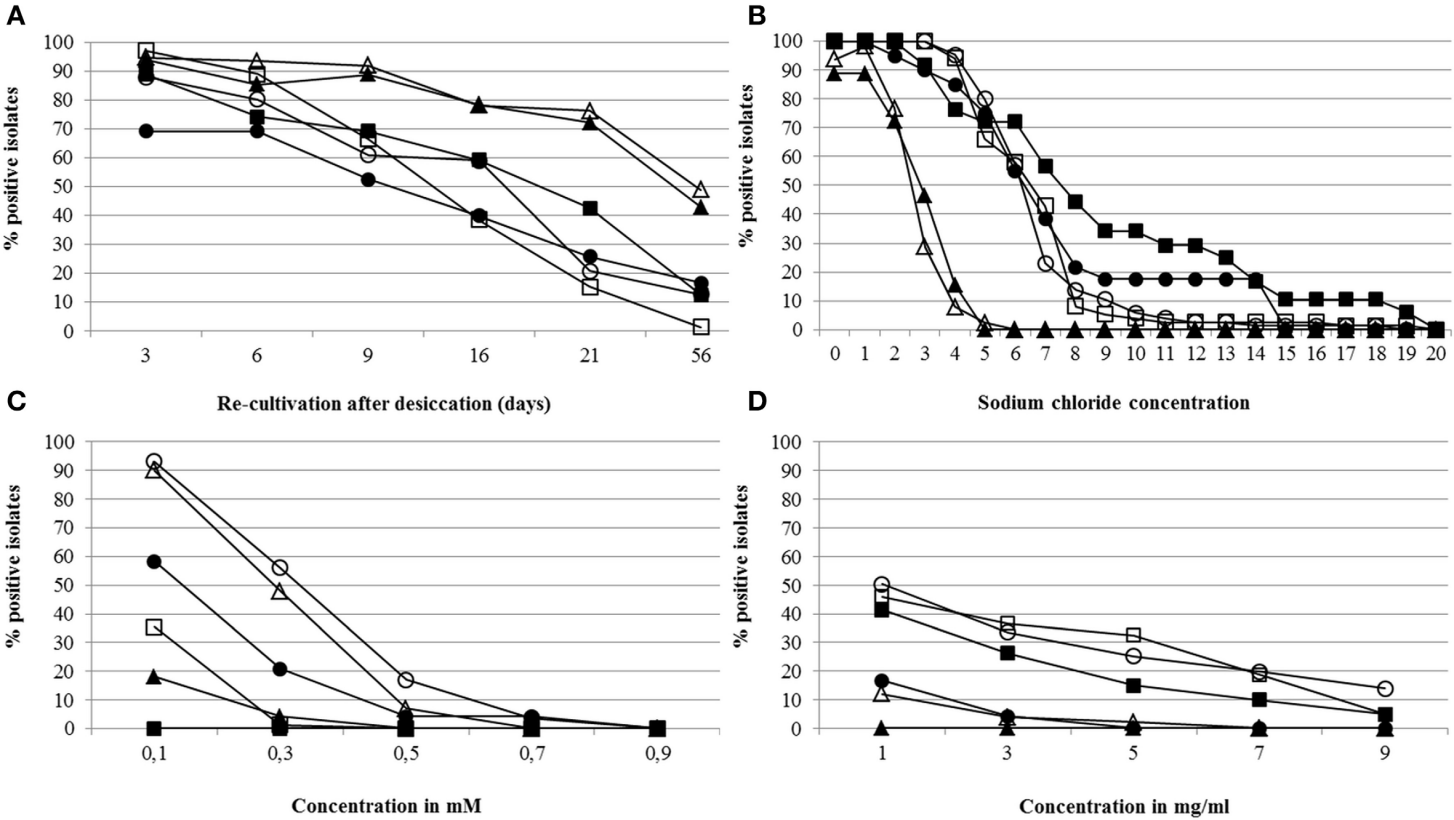
**Stress tolerance assays**. Bacterial isolates able to be re-cultivated after desiccation **(A)**, grown in presence of different sodium chloride concentrations **(B)**, and cultivated in presence of reactive oxygen species caused by hydrogen peroxide **(C)** and tellurite **(D)**. In total, 357 antagonistic bacteria, previously selected from R2A or Kings B medium, were tested. Legend: bacteria isolated from R2A—closed symbols, and from Kings B—open symbols; wild beet rhizosphere (WB-CS)—circle, rhizosphere of sugar beet plants cultivated in coastal drift line soil (SB-CS)—square, rhizosphere of sugar beet plants cultivated in potting soil (SB-PS)—triangle. Further statistics were indicated in Tables S3A–D.

All positively evaluated bacterial isolates (357) were further tested for their ability to tolerate stress including desiccation, salt, and reactive oxygen species caused by hydrogen peroxide and tellurite (Figure 3). In desiccation assays, the survival of the cells over several days was tested. In general, slightly more isolates obtained from the Kings B medium could be re-cultivated after 3, 6, and 16 days when compared to R2A. After 56 days, most of the isolates were not able to be re-cultivated after desiccation (Figure 3A). In salt (NaCl) stress assays, a high percentage of isolates from WB-CS and sugar beet plants cultivated in the same soil (SB-CS) were able to grow in the presence of up to 6% salt. The majority of bacteria from all samples could deal with a maximal salt concentration of 6%; with the exception of SB-PS, where most of the isolates were unable to grow above 2% NaCl (Figure 3B). In the presence of reactive oxygen species (ROS) induced by hydrogen peroxide and tellurite, 68.0 and 28.2%, respectively, of the antagonists were able to cope with the lowest concentration, and 1.1 and 4.5%, respectively, with the highest concentration. The highest percentage of isolates able to deal with high ROS concentrations caused by hydrogen peroxide were determined for isolates from WB-CS (0.1 mM: 91.7%; 0.3 mM: 53.3%; 0.5 mM: 16.7%) selected on Kings B (Figure 3C). The maximum concentration of hydrogen peroxide at which isolates were able to grow was 0.7 mM. None of the isolates from SB-CS and from Kings B were able to grow in the presence of H_2_O_2_. Among all samples, only a few isolates were able to tolerate high ROS concentrations from tellurite. These isolates were obtained from SB-CS. The maximum concentration of tellurite at which isolates were able to grow was 9 mg ml^−1^ (Figure 3D).

Bacterial isolates with the highest antagonistic activity and stress tolerance were identified (Table 4). From all samples, isolates of *Pseudomonas* and *Stenotrophomonas* species were identified. Isolates from the sugar beet rhizospheres (SB-CS, SB-PS) were mainly identified as *Staphylococcus* and *Stenotrophomonas* species. In contrast, from WB rhizospheres more diverse bacterial species were isolated, i.e., *Curtobacterium* sp., *Erwinia* sp., *Microbacterium* sp., *Micrococcus* sp., *Streptomyces* sp., *Sphingobacterium* sp., *Agrobacterium* sp., *Luteimonas* sp., *Pseudomonas* sp., *Rheinheimera* sp., and *Stenotrophomonas* sp. Based on their 16S rRNA gene similarity, the abundance of bacterial isolates in the amplicon libraries was calculated. In all samples, 16S rRNA sequences were detected of species to which the active isolates were affiliated. Their relative abundances ranged from 0.01 to 2.94% with the highest abundances of *Pseudomonas* and *Stenotrophomonas*.

**Table 4:** **Taxonomic classification of the most active antagonists isolated from R2A (universal bacteria) and Kings B (selective for *Pseudomonadaceae*) by sequencing partial 16S rRNA genes**.

**Antagonist**	**Identification**				**Antagonistic activity**					**Stress tolerance^c^**			
	**Closest BLAST database match (reference RNA sequences)**	**SI in %^a^**	**Accession no**.	**Relative abundances (%)^b^**	***A. a.***	***B. c.***	***R. s.***	***S. s.***	***V. d.***	**Dry-off**	**NaCl**	**H_2_O_2_ [μmol]**	**Tellurit [mg/ml]**
**WILD BEET PLANTS GROWN IN COASTAL DRIFT LINE SOIL—WB-CS (R2A)**													
ZR1-24	*Micrococcus luteus*	99	NR_075062.1	No match	−	−	−	−	+	56	7	300	0
ZR3-3	*Rheinheimera aquimaris*	98	NR_044068.1	0.38	−	+	−	−	−	1	8		
ZR3-4	*Agrobacterium fabrum*	97	NR_074266.1	No match	−	−	−	+	−	21	5	100	0
ZR3-9	*Curtobacterium flaccumfaciens* pv. *flaccumfaciens*	99	NR_025467.1	0.01	+	−	−	−	−	56	7	700	0
ZR3-18	*Sphingobacterium faecium*	99	NR_025537.1	No match	−	+	−	−	−	16	5	100	0
ZR4-2	*Luteimonas aestuarii*	98	NR_044343.1	0.56	+	−	−	−	n. d.	9	15	0	0
ZR4-6	*Streptomyces phaeochromogenes*	98	NR_041200.1	No match	+	−	−	−	−	1	5	0	0
ZR4-9	*Microbacterium aoyamense*	99	NR_041332.1	No match	+	−	−	−	−	21	3	0	0
**WILD BEET PLANTS GROWN IN COASTAL DRIFT LINE SOIL—WB-CS (KINGS B)**													
ZR1-2_Ps	*Stenotrophomonas rhizophila*	99	NR_028930.1	0.04	−	+	−	−	−	6	19	0	0
ZR1-10_Ps	*Pseudomonas poae*	99	NR_102514.1	0.54	−	−	+	−	−	56	7	300	0
ZR2-5_Ps	*Stenotrophomonas rhizophila*	99	NR_028930.1	0.04	−	+	−	−	−	16	14	100	1
ZR2-9_Ps	*Stenotrophomonas rhizophila*	99	NR_028930.1	0.04	−	+	−	−	−	21	9	100	0
ZR3-2_Ps	*Erwinia rhapontici*	99	NR_041976.1	0.14	−	+	−	−	−	56	11	300	0
ZR3-5_Ps	*Pseudomonas brassicacearum* subsp. *brassicacearum*	99	NR_074834.1	0.03	−	+	−	−	−	56	7	300	9
ZR3-8_Ps	*Erwinia rhapontici*	99	NR_041976.1	0.14	−	+	−	−	−	56	9	300	0
ZR4-7_Ps	*Pseudomonas poae*	99	NR_102514.1	0.54	+	+	+	−	−	56	5	700	1
ZR4-10_Ps	*Stenotrophomonas maltophilia*	99	NR_074875.1	0.39	+	−	−	−	−	56	8	300	0
ZR4-23_Ps	*Pseudomonas libanensis*	99	NR_024901.1	0.54	+	+	−	+	+	16	8	100	1
**SUGAR BEET PLANTS CULTIVATED IN COASTAL DRIFT LINE SOIL—SB-CS (R2A)**													
VN1/1-7	*Staphylococcus epidermidis*	99	NR_036904.1	No match	+	+	−	+	−	16	20	0	3
VN1/2-9	*Staphylococcus epidermidis*	99	NR_036904.1	No match	−	+	−	−	−	3	14	0	0
VN1/2-11	*Microbacterium paraoxydans*	98	NR_025548.1	No match	+	−	−	−	−	16	8	0	0
VN2/1-12	*Stenotrophomonas rhizophila*	99	NR_028930.1	No match	+	+	−	−	−	9	11	0	0
VN3/1-8	*Micrococcus luteus*	99	NR_075062.1	No match	−	+	−	−	−	21	5	0	1
**SUGAR BEET PLANTS CULTIVATED IN COASTAL DRIFT LINE SOIL—SB-CS (KINGS B)**													
VN1/1-2_Ps	*Stenotrophomonas rhizophila*	99	NR_028930.1	No match	−	−	−	−	+	56	10	0	0
VN1/1-5_Ps	*Staphylococcus epidermidis*	99	NR_036904.1	No match	+	−	−	+	+	21	11	300	3
VN1/2-9_Ps	*Stenotrophomonas maltophilia*	98	NR_074875.1	No match	−	−	−	−	+	21	6	0	0
VN1/2-11_Ps	*Stenotrophomonas rhizophila*	99	NR_028930.1	No match	−	−	−	+	+	16	7	0	0
VN1/2-12_Ps	*Stenotrophomonas rhizophila*	99	NR_028930.1	No match	−	−	−	+	+	21	7	0	0
VN3/1-1_Ps	*Pseudomonas brassicacearum* subsp. *brassicacearum*	99	NR_074834.1	No match	+	+	−	+	+	6	8	100	7
VN3/1-8_Ps	*Pseudomonas brassicacearum* subsp. *brassicacearum*	99	NR_074834.1	No match	−	+	−	+	+	16	8	100	7
VN4/1-1_Ps	*Pseudomonas mosselii*	99	NR_024924.1	0.18	+	+	+	+	+	9	8	100	5
VN4/1-2_Ps	*Pseudomonas gessardii*	99	NR_024928.1	No match	+	+	+	+	+	9	8	100	7
**SUGAR BEET PLANTS CULTIVATED IN POTTING SOIL—SB-PS (R2A)**													
VS2/3-9	*Arthrobacter ilicis*	99	NR_104950.1	0.21	−	+	−	−	n. d.	56	4	0	0
VS2/3-10	*Stenotrophomonas rhizophila*	99	NR_028930.1	0.43	−	+	−	−	n. d.	56	5	100	0
VS2/4-8	*Stenotrophomonas rhizophila*	99	NR_028930.1	0.38	−	+	−	−	n. d.	56	4	0	0
VS3/1-7	*Stenotrophomonas rhizophila*	99	NR_028930.1	0.43	−	+	−	−	n. d.	56	5	0	0
VS3/2-2	*Stenotrophomonas rhizophila*	99	NR_028930.1	0.43	−	+	−	−	n. d.	56	5	0	0
VS3/2-9	*Stenotrophomonas rhizophila*	99	NR_028930.1	0.43	−	+	−	−	n. d.	56	4	0	0
**SUGAR BEET PLANTS CULTIVATED IN POTTING SOIL—SB-PS (KINGS B)**													
VS1/1-2_Ps	*Pseudomonas veronii*	99	NR_028706.1	2.82	−	+	−	n. d.	n. d.	56	5	300	1
VS1/1-9_Ps	*Stenotrophomonas rhizophila*	99	NR_028930.1	0.43	−	+	−	n. d.	n. d.	56	3	500	0
VS1/2-8_Ps	*Stenotrophomonas rhizophila*	99	NR_028930.1	0.43	−	+	−	n. d.	n. d.	56	3	300	0
VS1/3-5_Ps	*Stenotrophomonas rhizophila*	99	NR_028930.1	0.43	−	+	−	n. d.	n. d.	56	4	100	0
VS1/3-7_Ps	*Stenotrophomonas rhizophila*	99	NR_028930.1	No match	−	+	−	n. d.	n. d.	56	4	100	0
VS1/4-5_Ps	*Stenotrophomonas rhizophila*	99	NR_028930.1	0.43	−	+	−	n. d.	n. d.	56	4	100	0
VS2/1-7_Ps	*Stenotrophomonas rhizophila*	99	NR_028930.1	0.43	−	+	−	n. d.	n. d.	56	4	100	0
VS2/2-6_Ps	*Yersinia kristensenii*	99	NR_025159.1	0.01	−	−	+	n. d.	n. d.	56	5	300	0
VS2/4-4_Ps	*Pseudomonas oryzihabitans*	99	NR_025881.1	0.21	−	+	−	n. d.	n. d.	56	5	100	0
VS2/4-7_Ps	*Stenotrophomonas rhizophila*	99	NR_028930.1	0.43	−	+	−	n. d.	n. d.	56	3	300	0
VS2/4-8_Ps	*Stenotrophomonas rhizophila*	99	NR_028930.1	0.38	−	+	−	n. d.	n. d.	56	3	300	0
VS2/4-9_Ps	*Stenotrophomonas rhizophila*	99	NR_028930.1	0.43	−	+	−	n. d.	n. d.	56	3	300	0
VS3/1-5_Ps	*Stenotrophomonas rhizophila*	99	NR_028930.1	0.38	−	+	+	n. d.	n. d.	56	3	100	0
VS3/2-3_Ps	*Pseudomonas brenneri*	99	NR_025103.1	2.70	−	+	−	n. d.	n. d.	9	5	500	1
VS3/3-4_Ps	*Pseudomonas putida*	99	NR_074739.1	0.11	−	+	−	n. d.	n. d.	9	6	500	1
VS3/3-9_Ps	*Stenotrophomonas rhizophila*	99	NR_028930.1	0.43	−	+	−	n. d.	n. d.	56	3	300	0
VS3/4-1_Ps	*Pseudomonas lurida*	100	NR_042199.1	2.94	+	+	+	n. d.	n. d.	21	5	500	3
VS4/1-5_Ps	*Stenotrophomonas rhizophila*	99	NR_028930.1	0.43	−	+	−	n. d.	n. d.	56	3	300	0
VS4/3-2_Ps	*Pseudomonas costantinii*	99	NR_025164.1	2.94	−	−	+	n. d.	n. d.	56	4	300	1
VS4/3-10_Ps	*Stenotrophomonas maltophilia*	99	NR_074875.1	0.01	−	+	−	n. d.	n. d.	56	4	300	0
VS4/4-5_Ps	*Stenotrophomonas maltophilia*	99	NR_074875.1	0.01	−	+	−	n. d.	n. d.	21	3	500	0
VS4/4-10_Ps	*Pseudomonas veronii*	99	NR_028706.1	2.82	−	+	−	n. d.	n. d.	21	4	500	1

Sequences from the isolates were aligned with representative sequences of OTUs from amplicon sequencing data set.

a Similarity index (SI) for isolates identified by partial 16S rRNA gene sequencing.

b Relative abundances (%) of OTUs with at least 99% similarity were considered and indicated for OTUs in corresponding samples.

c Maximum concentration of bacterial isolates able to be re-cultivated after desiccation (dry-off), to grow at maximum concentration of sodium chloride concentrations (NaCl), hydrogen peroxide (H_2_O_2_) and tellurite. n. d.—not determined.

## Discussion

In this study, we found differences within the microbiome composition of the sugar beet rhizosphere and its wild ancestor. At phylum level, the rhizosphere microbiome of wild beet and domesticated sugar beet plants (WB-CS, SB-CS) resembled each other independently from the climate and plant development, whereas, at a higher taxonomic resolution plant genotype-specific patterns were identified. This plant genotype-specific effect was confirmed for functional traits. The wild beet rhizosphere (WB-CS) was colonized by a low number of bacteria with antagonistic activity against pathogens but their antagonists showed a high potential to cope with abiotic stresses. Conversely, the sugar beet cultivar was able to enrich a high antagonistic potential from both soil types but harbored less antagonists with high stress resistance. In addition, an impact of both soil types (CS, PS) on the bacterial composition was found. However, the differences in structure and function of the microbiomes of ancient and modern beets underline the relationship between the plant genotypes and their associated bacteria and confirmed our hypotheses.

The composition of the microbiome of both plant genotypes, when grown in CS, was comparable at phylum level. However, resolved at OTU level, based on results from molecular fingerprint analysis and amplicon pyrosequencing of 16S rRNA genes, plant genotype-specific OTUs were detected. The wild beet (WB-CS) showed the highest number of unique bands in bacterial fingerprints but the bacterial communities in the rhizosphere of sugar beet (SB-CS) and in bulk soil (CS) were similar to each other. Based on amplicon libraries, the WB-CS rhizosphere consisted of 18 unique out of 38 detected OTUs including *Flavobacterium*, *Pedobacter*, and *Pseudomonas* spp. In contrast, no specific OTUs could be identified for SB-CS. The low rhizosphere effect in SB-CS might be a result of the shorter growth period in addition to the different climate. The majority of the dominant OTUs (20) was shared by SB-CS and WB-CS and included genera of the phyla *Actinobacteria* (*Thermoleophilum*), *Proteobacteria* (*Cystobacter*, *Desulfomonile*), and *Planctomycetes* (*Blastopirellula*). In all rhizospheres (CS), the myxobacterium *Angiococcus disciformis* was found in highest relative abundances. The more diverse spectrum of OTUs associated with wild beet plants compared to SB-CS was confirmed by the elevated Shannon index, which was 8.7 for WB-CS and 8.1 for SB-CS. The impact of the plant genotype on the associated microbiota, which was clearly shown in our study, was dependent on the applied method and their taxonomic resolution. This genotype-specific impact was also shown or other crops such as maize (Peiffer et al., 2013), potato (Weinert et al., 2009, 2011), and rice (Engelhard et al., 2000; Hardoim et al., 2011).

In addition to the plant genotype-specific community structures, we found differences in *in vitro* functions of the isolates as well. The proportion of strains with antagonistic *in vitro* activity against phytopathogens was lower in the rhizosphere WB-CS in comparison to the domesticated sugar beet SB-CS and SB-PS. The lower antagonistic activity of wild beet-associated bacteria can be traced back to the fact that pathogen pressure barely exists in coastal drift-line soil as it is influenced by seawater spray, tidal flow, and storms (Biancardi et al., 2012). In contrast, modern sugar beets are threatened by many diseases including fungal pathogens which were involved in this study (Khan, 2013). Under agricultural conditions, the pathogen pressure is often much higher than in natural habitats; one reason for this can be the naturally occurring biodiversity which protects ecosystems from spread of diseases (Latz et al., 2012). *B. maritima* grows under extreme saltwater conditions, and can tolerate both high salt concentrations in soil and severe drought (Shaw et al., 2002). Wild beet-associated bacterial isolates showed a higher extend of stress tolerance than isolates of domesticated sugar beet plants with *in vitro* antagonistic activity. These isolates were identified mainly as *Pseudomonas* and *Stenotrophomonas* species; members of both are well-known for their ability to cope with biotic and abiotic stress (Alavi et al., 2013; Zachow et al., 2013). Within the amplicon libraries their relative abundances was lower than 1%. However, the protective functions can be fulfilled by a minor fraction, which may contribute to important ecosystem functions (Pester et al., 2010).

The community composition of both sugar beet genotypes showed clear soil type-specific effects. In bacterial fingerprints, SB-CS showed only 36% similarity to that of SB-PS. Based on the pyrosequencing approach, the phyla *Actinobacteria* and *Planctomycetes* were pin-pointed as determinants for these differences. These taxa were found exclusively in WB-CS and SB-CS. *Planctomycetes* are known as soil oligotrophes and were enriched in the bulk soil compared to the maize rhizosphere (Peiffer et al., 2013). *Bacteroidetes* and *Proteobacteria* were important members of the rhizosphere community. At OTU level the most dominant OTUs belong to the genus *Pseudomonas*. Sugar beets were previously reported as highly colonized by *Pseudomonas* species using a broad variety of techniques (Lambert et al., 1990; Thrane et al., 2000; Zachow et al., 2008; Mendes et al., 2011). In the current study, *Pseudomonas* species were detected within all bacterial communities using fingerprint and pyrosequencing analysis. In bacterial fingerprints, *Pseudomonas* species were mainly detected in samples grown in PS and were less dominant in the rhizosphere samples cultivated CS. Interestingly, pseudomonads appears to have a minor relevance for the investigated *Beta* genotypes grown or cultivated in their natural habitat.

In this study, we compared the microbiome of both ancestral and domesticated beet rhizospheres and linked functions to particular isolates within the bacterial community. These results support the suggestion by Wissuwa et al. (2009) to apply the knowledge of plant genotype-specific traits of associated microorganisms for breeding strategies. Moreover, wild types of crops as well as soil from their original distribution area are important sources for isolates with antagonistic activity against plant pathogens or with stress protecting activity for their hosts.

## Author contributions

Christin Zachow, Henry Müller, Gabriele Berg conceived and guided the research, and wrote the manuscript. Ralf Tilcher provided experimental suggestions. Christin Zachow, Henry Müller performed the laboratory experiments and bioinformatoric analyses.

### Conflict of interest statement

The authors declare that the research was conducted in the absence of any commercial or financial relationships that could be construed as a potential conflict of interest.
