# Supplementary material for: Differences between the rhizosphere microbiome of Beta vulgaris ssp. maritima—ancestor of all beet crops—and modern sugar beets
Source: Front Microbiol. 2014 Aug 26;5:415. doi: 10.3389/fmicb.2014.00415 (PMC4144093; doi:10.3389/fmicb.2014.00415)
Supplement: Supplementary file 1 [file Presentation1.PDF]

Table S1 Pyrosequence primers: adapter (italic), linker (bold), and sample-specific tags of 10 nucleotides (underlined) used for 454 pyrosequencing in this study.

Primer label	Primer sequence *ADAPTER*–**LINKER**–tag–PRIMER
unibacII515f_MID1: WB-CS	*CGTATCGCCTCCCTCGCGCCA***TCAG**acgagtgcgtGTGCCAGCAGCCGC
unibacII515f_MID2: CS	*CGTATCGCCTCCCTCGCGCCA***TCAG**acgctcgacaGTGCCAGCAGCCGC
unibacII515f_MID3: SB-CS	*CGTATCGCCTCCCTCGCGCCA***TCAG**agacgcactcGTGCCAGCAGCCGC
unibacII515f_MID4: SB-PS	*CGTATCGCCTCCCTCGCGCCA***TCAG**agcactgtagGTGCCAGCAGCCGC
unibacII515f_MID5: PS	*CGTATCGCCTCCCTCGCGCCA***TCAG**atcagacacgGTGCCAGCAGCCGC

Table S2 Relative abundance (%) of genera in the rhizosphere of wild beet plants (WB-CS), sugar beet plants cultivated in coastal drift line soil or potting soil (SB-CS, SB-PS) and compared to the corresponding bulk soil (CS, PS) based on pyrosequencing data. Positive rhizosphere and soil responders were indicated in the ratio column by green. Red colored cells in the ration columns indicate a negative response of the particular taxa. Non-detectable taxa in respective samples are expressed by n. d.

Phylum	***Genus***	***WB-CS***	***CS***	***SB-CS***	***Ratio WB-CS/CS***	***Ratio SB-CS/CS***	***SB-PS***	***PS***	***Ratio SB-PS/PS***
Other phyla		8.58	13.60	15.02	0.63	1.10	1.46	0.56	2.61
Acidobacteria	*Other*	7.67	13.33	10.11	0.58	0.76	0.03	0.00	n. d. in PS
	Candidatus Solibacter	1.08	3.74	2.87	0.29	0.77	0.03	0.00	n. d. in PS
Actinobacteria	*Other*	16.70	5.90	5.15	2.83	0.87	1.44	1.48	0.97
	Aeromicrobium	0.10	0.01	0.00	8.75	n. d. in SB-CS	0.03	0.00	n. d. in PS
	Arthrobacter	0.27	0.02	0.00	11.38	n. d. in SB-CS	0.67	0.00	n. d. in PS
	Demequina	0.25	0.05	0.00	5.25	n. d. in SB-CS	0.00	0.00	n. d.
	Frigoribacterium	0.03	0.00	0.02	n. d. in CS	n. d. in CS	0.10	0.00	n. d. in PS
	Iamia	0.12	0.04	0.04	3.50	1.02	0.00	0.00	n. d.
	Microbacterium	0.60	0.02	0.00	25.82	n. d. in SB-CS	0.18	0.00	n. d. in PS
	Microlunatus	0.15	0.00	0.00	n. d. in CS	n. d. in CS	0.00	0.00	n. d.
	Mycobacterium	0.10	0.00	0.02	n. d. in CS	n. d. in CS	0.00	0.01	n. d. in SB-PS
	Pseudonocardia	0.11	0.00	0.02	n. d. in CS	n. d. in CS	0.00	0.85	n. d. in SB-PS
	Salinibacterium	0.12	0.01	0.00	10.50	n. d. in SB-CS	0.05	0.01	4.76
	Streptomyces	0.32	0.06	0.09	5.43	1.53	0.00	0.09	n. d. in SB-PS
Bacteroidetes	*Other*	1.79	0.74	0.50	2.43	0.68	2.03	0.04	46.37
	Pedobacter	0.08	0.00	0.00	n. d. in CS	n. d.	5.95	0.00	n. d. in PS
	Sphingobacterium	0.01	0.00	0.00	n. d. in CS	n. d.	0.93	0.49	1.88
	Flavobacterium	0.78	0.00	0.00	n. d. in CS	n. d.	5.21	0.46	11.32
Firmicutes	*Other*	0.37	0.07	0.14	5.25	2.04	0.24	4.26	0.06
	Alicyclobacillus	0.01	0.00	0.02	n. d. in CS	n. d. in CS	0.48	3.80	0.13
	Bacillus	1.00	0.08	0.07	12.26	0.88	0.29	7.98	0.04
	Ammoniphilus	0.00	0.00	0.00	n. d.	n. d.	0.01	1.18	0.01
	Brevibacillus	0.00	0.00	0.00	n. d.	n. d.	0.03	2.62	0.01

Phylum	***Genus***	***WB-CS***	***CS***	***SB-CS***	***Ratio WB-CS/CS***	***Ratio SB-CS/CS***	***SB-PS***	***PS***	***Ratio SB-PS/PS***
	Cohnella	0.00	0.00	0.04	n. d. in CS	n. d. in CS	0.17	2.68	0.06
	Paenibacillus	0.52	0.06	0.04	8.93	0.61	0.49	11.92	0.04
	Sporosarcina	0.16	0.04	0.02	4.67	0.51	0.14	2.51	0.05
Planctomycetes	*Other*	17.59	27.20	27.97	0.65	1.03	0.18	0.00	n. d. in PS
	Gemmata	1.59	0.62	0.91	2.56	1.47	0.02	0.00	n. d. in PS
	A17	1.96	2.30	2.81	0.85	1.22	0.01	0.00	n. d. in PS
	Pirellula	0.74	0.61	0.90	1.21	1.47	0.02	0.00	n. d. in PS
	Planctomyces	5.06	5.57	6.70	0.91	1.20	0.50	0.00	n. d. in PS
Proteobacteria	*Other*	18.48	8.09	6.56	2.28	0.81	47.96	22.00	2.18
	Achromobacter	0.00	0.01	0.00	n. d. in WB-CS	n. d. in SB-CS	0.16	7.17	0.02
	Acinetobacter	0.00	0.00	0.00	n. d.	n. d.	0.69	0.00	n. d. in PS
	Agrobacterium	0.15	0.00	0.02	n. d. in CS	n. d. in CS	0.34	0.00	n. d. in PS
	Amaricoccus	0.30	0.04	0.04	8.46	1.02	0.01	0.00	n. d. in PS
	Aminobacter	0.16	0.00	0.00	n. d. in CS	n. d.	0.09	0.00	n. d. in PS
	Aquimonas	0.10	0.18	0.75	0.58	4.29	0.00	0.00	n. d.
	Arenimonas	0.09	0.13	0.04	0.72	0.28	0.00	0.00	n. d.
	Azomonas	0.00	0.00	0.00	n. d.	n. d.	0.16	0.00	n. d. in PS
	BD2-13	0.14	0.00	0.00	n. d. in CS	n. d.	0.03	0.00	n. d. in PS
	Bdellovibrio	0.03	0.04	0.00	0.88	n. d. in SB-CS	0.31	0.00	n. d. in PS
	Bosea	0.03	0.00	0.00	n. d. in CS	n. d.	0.23	1.91	0.12
	Bradyrhizobium	0.08	0.02	0.04	3.50	1.53	0.04	0.19	0.22
	Brevundimonas	0.05	0.00	0.00	n. d. in CS	n. d.	1.40	0.00	n. d. in PS
	Candidatus Entotheonella	10.33	17.89	18.93	0.58	1.06	0.00	0.00	n. d.
	Caulobacter	0.02	0.00	0.00	n. d. in CS	n. d.	0.42	0.00	n. d. in PS
	Cellvibrio	0.77	0.02	0.14	32.83	6.13	1.61	0.00	n. d. in PS
	Devosia	0.92	0.11	0.07	8.75	0.68	2.66	0.00	n. d. in PS
	Dokdonella	0.67	0.05	0.02	14.22	0.38	0.02	0.00	n. d. in PS
	Hydrogenophaga	0.28	0.01	0.02	23.64	1.53	0.48	0.00	n. d. in PS

Phylum	***Genus***	***WB-CS***	***CS***	***SB-CS***	***Ratio WB-CS/CS***	***Ratio SB-CS/CS***	***SB-PS***	***PS***	***Ratio SB-PS/PS***
	Hyphomicrobium	0.25	0.07	0.05	3.50	0.77	0.00	0.00	n. d.
	Janthinobacterium	0.07	0.00	0.00	n. d. in CS	n. d.	3.83	0.00	n. d. in PS
	Kaistia	0.00	0.00	0.00	n. d.	n. d.	0.38	0.00	n. d. in PS
	Lutibacterium	0.07	0.01	0.00	6.13	n. d. in SB-CS	0.25	0.00	n. d. in PS
	Methylophaga	0.04	0.00	0.00	n. d. in CS	n. d.	0.10	0.00	n. d. in PS
	Methylotenera	0.30	0.00	0.04	n. d. in CS	n. d. in CS	1.70	0.00	n. d. in PS
	Novosphingobium	0.87	0.01	0.02	74.41	1.53	2.79	0.00	n. d. in PS
	Paracoccus	0.24	0.01	0.00	20.13	n. d. in SB-CS	1.04	0.00	n. d. in PS
	Pedomicrobium	0.07	0.04	0.11	2.04	3.07	0.00	0.00	n. d.
	Pigmentiphaga	0.04	0.00	0.00	n. d. in CS	n. d.	0.40	0.00	n. d. in PS
	Polaromonas	0.13	0.00	0.00	n. d. in CS	n. d.	0.00	0.00	n. d.
	Pseudomonas	0.59	0.01	0.00	50.77	n. d. in SB-CS	4.96	0.05	90.55
	Pseudoxanthomonas	0.33	0.00	0.09	n. d. in CS	n. d. in CS	0.29	0.18	1.66
	Rheinheimera	0.02	0.02	0.02	0.88	0.77	0.48	0.00	n. d. in PS
	Rhizobium	0.00	0.01	0.00	n. d. in WB-CS	n. d. in SB-CS	0.61	0.00	n. d. in PS
	Rhodoplanes	0.25	0.05	0.13	5.25	2.68	n. d.	0.00	n. d.
	Roseomonas	0.01	0.00	0.00	n. d. in CS	n. d.	0.10	0.00	n. d. in PS
	Shinella	0.01	0.00	0.00	n. d. in CS	n. d.	0.11	0.00	n. d. in PS
	Sphingobium	0.04	0.00	0.00	n. d. in CS	n. d.	0.45	0.00	n. d. in PS
	Sphingomonas	0.17	0.01	0.00	14.88	n. d. in SB-CS	1.27	0.01	116.04
	Sphingopyxis	0.16	0.01	0.00	14.01	n. d. in SB-CS	1.44	4.70	0.31
	Stenotrophomonas	0.39	0.00	0.00	n. d. in CS	n. d.	1.82	21.35	0.09
	Yersinia	0.01	0.00	0.00	n. d. in CS	n. d.	0.50	0.00	n. d. in PS
Verrucomicrobia	*Other*	1.19	0.84	0.90	1.41	1.06	0.38	0.00	n. d. in PS
	Opitutus	0.13	0.04	0.07	3.79	2.04	0.06	0.00	n. d. in PS
	Luteolibacter	0.34	0.00	0.02	n. d. in CS	n. d. in CS	0.29	0.00	n. d. in PS

Table S3A Percentage of isolates able to be re-cultivated after several days of desiccation. Different letters indicate significant differences (P≤0.05). B – bacteria (isolated on R2A), Ps – *Pseudomonadaceae* (isolated on Kings B).

Days post desiccation	***Percentage of positive isolates (%)***					
	WB-CS (B)	***WB-CS (Ps)***	***SB-CS (B)***	***SB-CS (Ps)***	***SB-PS (B)***	***SB-PS (Ps)***
3	69±15 a	88±5 a	89±9 a	97±4 a	94±9 a	95±5 a
6	69±15 a	80±5 a	74±15 a	89±9 a	85±12 a	93±6 a
9	53±4 a	61±7 a	69±15 a	66±17 a	89±9 a	92±7 a
16	40±19 a	59±13 a	59±24 a	39±19 a	78±22 a	78±15 a
21	26±15 a	21±5 a	43±27 a	15±12 a	72±20 a	76±14 a
56	17±17 a	13±6 a	13±18 a	1±2 a	43±26 a	49±24 a

Table S3B Positive isolates able to grow in presence of different sodium chloride concentrations. Different letters indicate significant differences (P≤0.05). B – bacteria (isolated on R2A), Ps – *Pseudomonadaceae* (isolated on Kings B).

Concentration of NaCl (%)	***Percentage of positive isolates (%)***					
	WB-CS (B)	***WB-CS (Ps)***	***SB-CS (B)***	***SB-CS (Ps)***	***SB-PS (B)***	***SB-PS (Ps)***
0	100±0 a	100±0 a	100±0 a	100±0 a	89±9 a	94±9 a
1	100±0 a	100±0 a	100±2 a	100±0 a	89±9 a	98±2 a
2	95±7 a	100±0 a	100±0 a	100±0 a	72±20 a	77±9 a
3	90±14 c	100±0 c	92±12 c	100±0 c	46±31 ab	29±3 a
4	85±21 b	95±4 b	76±5 b	94±8 b	15±8 a	8±3 a
5	75±27 b	80±12 b	72±5 b	66±14 b	0±0 a	2±2 a
6	55±23 b	57±21 b	72±5 b	58±8 b	0±0 a	0±0 a
7	38±10 a	23±7 a	57±6 a	43±12 a	0±0 a	0±0 a
8	22±15 a	14±7 a	44±9 a	8±9 a	0±0 a	0±0 a
9	18±17 a	10±5 a	34±9 a	5±7 a	0±0 a	0±0 a
10	18±17 a	6±3 a	34±9 a	4±6 a	0±0 a	0±0 a
11	18±17 a	4±4 a	29±15 a	3±4 a	0±0 a	0±0 a
12	18±17 a	3±2 a	29±15 a	3±4 a	0±0 a	0±0 a
13	18±17 a	3±2 a	25±15 a	3±4 a	0±0 a	0±0 a
14	18±17 a	1±2 a	17±17 a	3±4 a	0±0 a	0±0 a
15	0±0 a	1±2 a	10±9 a	3±4 a	0±0 a	0±0 a
16	0±0 a	1±2 a	10±9 a	3±4 a	0±0 a	0±0 a
17	0±0 a	1±2 a	10±9 a	1±2 a	0±0 a	0±0 a
18	0±0 a	1±2 a	10±9a	1±2 a	0±0 a	0±0 a
19	0±0 a	0±0 a	6±9 a	1±2 a	0±0 a	0±0 a
20	0±0 a	0±0 a	0±0 a	0±0 a	0±0 a	0±0 a

Table S3C Positive isolates able to grow in presence of different concentrations of hydrogen peroxide (H_2O_2). Different letters indicate significant differences ($P \leq 0.05$). B – bacteria (isolated on R2A), Ps – *Pseudomonadaceae* (isolated on Kings B).

Concentration of H_2O_2 (mmol)	***Percentage of positive isolates (%)***					
	WB-CS (B)	***WB-CS (Ps)***	***SB-CS (B)***	***SB-CS (Ps)***	***SB-PS (B)***	***SB-PS (Ps)***
100	58±13 bcd	93±7 d	0±0 a	35±15 abc	18±10 ab	90±8 cd
300	21±18 ab	56±16 b	0±0 a	1±2 a	4±6 a	48±6 b
500	4±6 a	17±9 a	0±0 a	0±0 a	0±0 a	7±5 a
700	4±6 a	4±3 a	0±0 a	0±0 a	0±0 a	0±0 a
900	0±0 a	0±0 a	0±0 a	0±0 a	0±0 a	0±0 a

Table S3D Positive isolates able to grow in presence of different concentrations of tellurite. Different letters indicate significant differences ($P \leq 0.05$). B – bacteria (isolated on R2A), Ps – *Pseudomonadaceae* (isolated on Kings B).

Concentration of tellurite mg ml^{-1} (mmol)	***Percentage of positive isolates (%)***					
	WB-CS (B)	***WB-CS (Ps)***	***SB-CS (B)***	***SB-CS (Ps)***	***SB-PS (B)***	***SB-PS (Ps)***
1 (3.9)	17±17 a	50±13 a	41±25 a	46±11 a	0±0 a	12±10 a
3 (11.8)	4±6 a	34±21 a	26±13 a	37±14 a	0±0 a	4±3 a
5 (19.7)	0±0 a	25±15 a	15±14 a	32±17 a	0±0 a	2±2 a
7 (27.6)	0±0 a	20±10 a	10±8 a	19±9 a	0±0 a	0±0 a
9 (35.5)	0±0 a	14±6 a	5±7 a	5±2 a	0±0 a	0±0 a

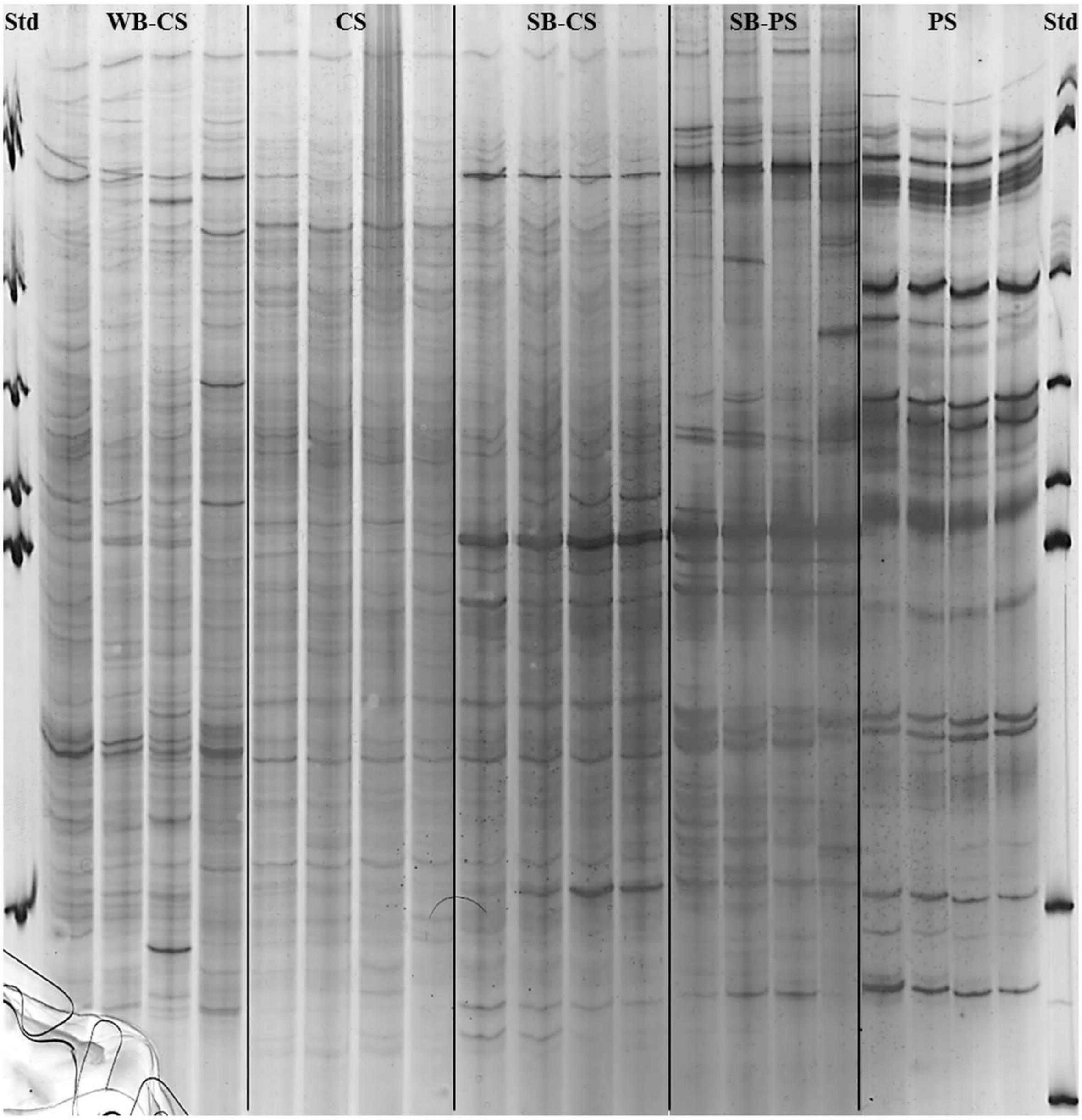

Figure S1 16S rRNA gene PCR-SSCP profiles of the bacterial communities in association with wild beet plants grown in coastal drift line soil (WB-CS) and rhizosphere of sugar beet plants cultivated in coastal drift line soil (SB-CS) and potting soil (SB-PS) and their respective bulk soils (CS and PS) with four repetitions per sample. Legend: Std – standard 1 kb ladder for gel analysis.

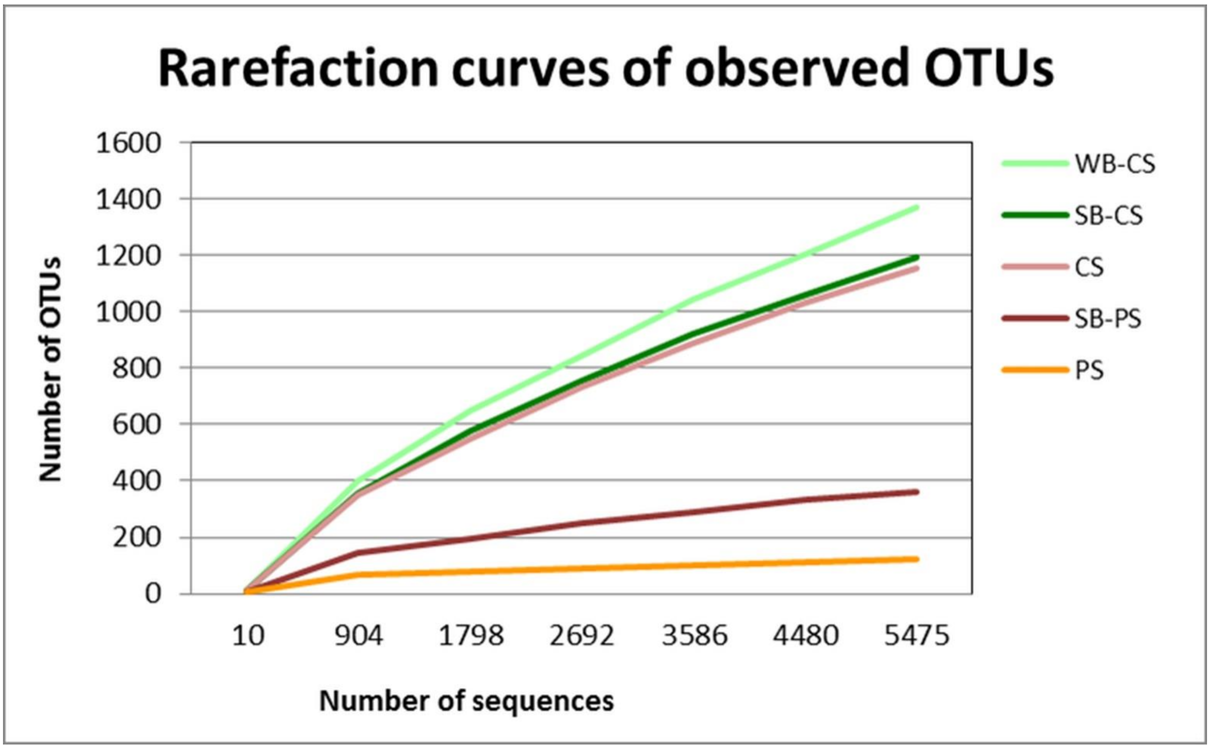

Figure S2 Rarefaction curves of observed OTUs at similarity level of 97%. The number of sequences for all samples was normalized to 5,578.
